# What is a cycling race simulation anyway: a review on protocols to assess durability in cycling

**DOI:** 10.1007/s00421-025-05725-1

**Published:** 2025-02-14

**Authors:** W. M. Peeters, M. Barrett, T. Podlogar

**Affiliations:** 1 School of Biomedical, Nutritional and Sport Sciences, Newcastle University, Newcastle-Upon-Tyne, UK; 2 Department of Public Health and Sport Sciences, University of Exeter Medical School, St Luke’s Campus, Exeter, UK; 3 School of Sport, Exercise and Rehabilitation Sciences, University of Birmingham, Birmingham, UK

**Keywords:** Durability, Cycling, Carbohydrates, Reliability, Pre-load, Performance

## Abstract

Physiological resilience or durability is now recognised as a determinant of endurance performance such as road cycling. Reliable, ecologically valid and standardised performance tests in laboratory-based cycling protocols have to be established to investigate mechanisms underpinning, and interventions improving durability. This review aims to provide an overview of available race simulation protocols in the literature and examines its rigour around themes that influence durability including (i) exercise intensity anchoring and (ii) carbohydrate intake whilst also (iii) inspecting reliability and justification of the developed protocols. Using a systematic search approach, 48 articles were identified that met our criteria as a cycling race simulation. Most protocols presented limitations to be recommended as exercise test to investigate durability, such as not appropriately addressing the influence of exercise intensity domains by anchoring exercise intensity as % peak power or % $\dot{\text{V}}$ O_2max_. Ten articles provided reliability data, but only one articles under the appropriate conditions. Most studies sufficiently controlled nutrition during trials but not in the days leading to the trials or just before the trials. Thus, there is a paucity in protocols that combine justification and reliability with optimal nutritional support and mimic the true demands of a road-cycling race. This review lists an overview of protocols that researchers could use with caution to select a protocol for future experiments, but encourages further development of improved protocols, including utilisation of virtual software applications.

**Supplementary Information:**

The online version contains supplementary material available at 10.1007/s00421-025-05725-1.

## Introduction

Cycling is a sport that consists of several competitive disciplines, such as track cycling, mountain-bike, para-cycling and bicycle motor cross, however the most common discipline is road cycling. Road cycling can be generally classed as an endurance sport where performance is conventionally determined by physiological parameters including maximal oxygen uptake capacity ($\dot{\text{V}}$ O_2max_), lactate thresholds (LT) and exercise economy/gross efficiency (Joyner and Coyle 2008). Whilst this applies for shorter events such as criteriums or time trials, results of longer events such as road races cannot be fully explained by these parameters. Recently a fourth dimension has been suggested to be a determinant for success in endurance exercise, which is defined as physiological resilience (Jones 2023), durability, fatigability or fatigue resistance; definitions which will be used interchangeably throughout the article. This is the ability to withstand the functional decline following acute and/or chronic stressor, in other words to limit a decline in an individual’s performance after accumulated work compared to the same performance conducted in a fresh state. Observational field-based studies demonstrate that this parameter distinguishes world-class athletes from their less successful peers, including observational maximal mean power profiles of under-23 vs. professional cyclist, ProTeam vs. WorldTour (Gallo et al. 2022; Mateo-March et al. 2022) and also within the WorldTour the successful riders are better able to cope with fatigue compared to their less successful peers (Erp et al. 2021).

The identification of the fatigue resistance concept as the fourth determinant of endurance performance will undoubtedly trigger research experiments to investigate the physiological mechanisms that make someone more resistant to fatigue (Hamilton et al. 2024; Stevenson et al. 2022; Barranco-Gil et al. 2024) and to find interventions to improve fatigue resistance, such as carbohydrate (CHO) ingestion to maintain CHO availability (Clark et al. 1985) or various training interventions (Matomäki et al. 2023). However, to make definitive conclusions about the mechanisms and effectiveness of interventions there is a need to establish reliable and ecologically valid performance tests, which are sensitive to changes in performance as ultimately this is the end-point of success (Currell and Jeukendrup 2008). In relation to road cycling, this means that the test–retest reliability of a performance test following a prolonged exercise protocol to accumulate work needs to be established so that subsequent experiments can use the coefficient of variation (CV) to establish the sample size necessary to test whether an intervention improves performance that is larger than the smallest worthwhile effect (Hopkins et al. 1999). Within road cycling this would mean that individuals would need to cycle for several hours before completing a performance test that is realistic to the performance during road races, whilst following optimal fuelling strategies (Thomas et al. 2016). Performance tests could include a short all-out sprint, or an uphill climb as these are scenarios where (elite) cycling road races are decided for winning a single race or stage or compete for overall general classification in multi-day stage races.

Notwithstanding several attempts being made to conduct scientific experiments outdoors on closed circuits (Heuberger et al. 2017; Klaris et al. 2024), due to logistics and challenges around standardisation, most experiments are forced to take place in a laboratory setting, limiting ecological validity. To partially overcome this limitation, attempts have been made to design laboratory-based cycling protocols to simulate the demands of a cycling road race on electronically or magnetically controlled cycling ergometers. For researchers, there is currently no one-stop overview of available protocols and there is currently no universally accepted or adopted protocol to simulate a cycling road race. Therefore, the objective of this review is to systematically search for existing cycling road race simulation protocols, evaluate the protocol against parameters known to influence durability, such as exercise intensity anchoring and nutritional support, evaluate markers of reliability and validity of the performance tests and provide recommendations on future directions.

## Methods and output

A detailed description of the systematic search performed to find relevant papers using a cycling race simulation protocol is available in Supplemental Material 1. Briefly, we searched for scientific articles that included a cycling protocol containing a “pre-load” of at least 90 min in duration, where the exercise intensity had to be variable or stochastic, followed by a performance test. Justification for this lower time boundary was to assume a reasonable probability to accumulate significant amount of work to induce metabolic perturbations that could affect fatigue, such as glycogen depletion (Hawley et al. 1997). The nature of the pre-load protocol had to include a variable or stochastic intensity protocol. Thus, articles were excluded if the exercise prior to the performance test was a fixed-intensity protocol (e.g. 2 h at 50% $\dot{\text{V}}$ O_2max_). The reason for this was that the ecological validity of fixed-intensity protocol is limited compared to power output profiles of road-cycling races (Sanders and Heijboer 2019). Moreover, performance has been shown to be differentially affected following a stochastic exercise pre-load compared to continuous fixed-intensity (Palmer et al. 1997; Leo et al. 2022). Information extracted from the protocols include a breakdown of the pre-load, performance test selection, carbohydrate intake information around the protocol, and information on reliability and justification where available.

**Fig. 1:**
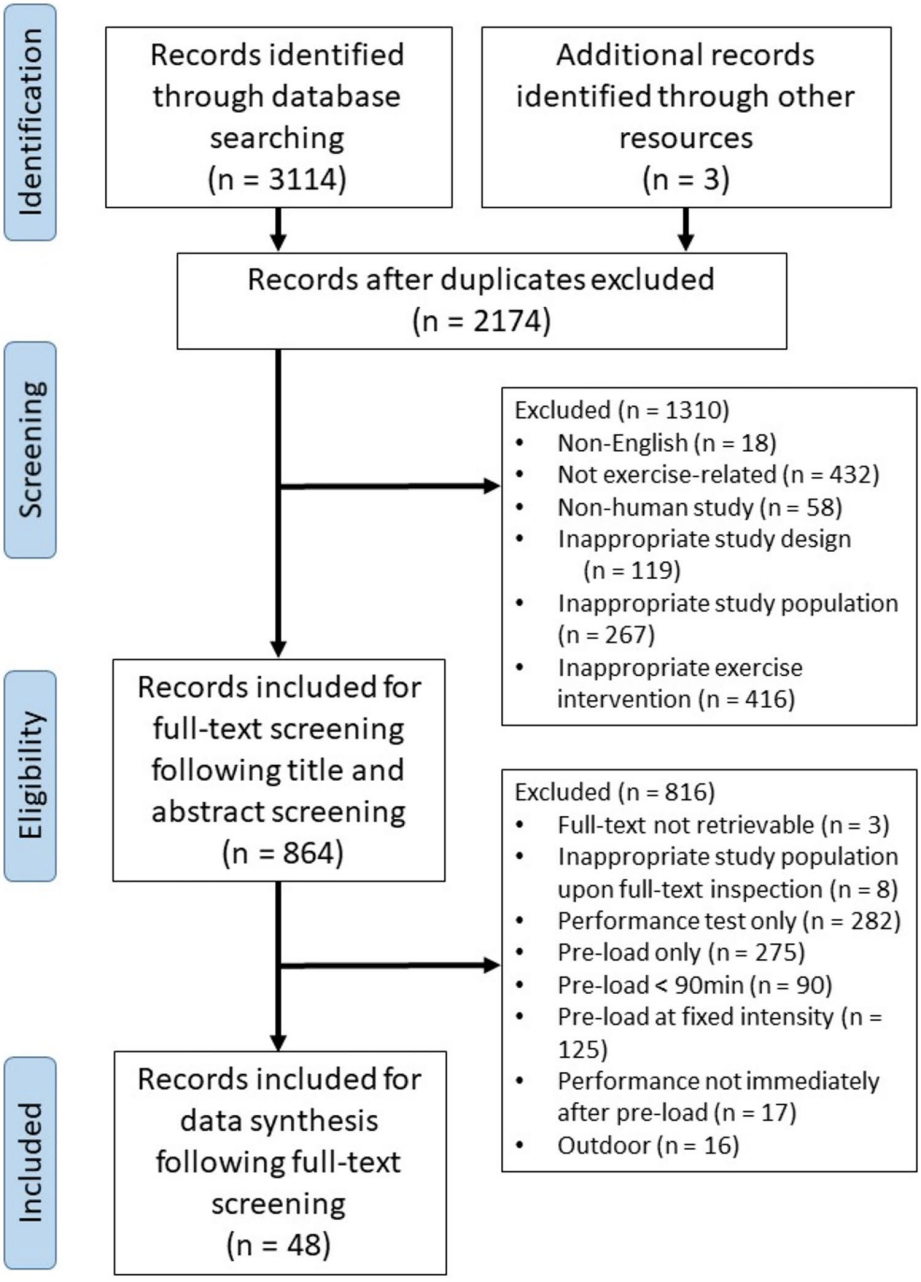
PRISMA flow diagram of articles with cycling race simulation protocols

A flowchart diagram is presented in Fig. 1. The initial search (7th January 2024) yielded 3114 articles. Following the removal of duplicates, 2171 articles remained and were screened for title and abstract on inclusion of a cycling-only exercise protocol in a relevant population. A total of 861 articles were included for further inspection on abstract and full-text to identify inclusion of an appropriate cycling exercise protocol (> 90 min “pre-load” of stochastic intensity followed by a performance test). Of these, 45 were included for data extraction. Two article was further identified by screening reference lists and one article was identified in the second search (30th April 2024). A total of 30 articles used a unique protocol, where 18 articles used one of the developed protocol in a subsequent study.

## Examination of exercise intensity selection in relation to durability

The influence of durability appears to present itself as a function of accumulated work over time and thus energy expenditure (Erp et al. 2021; Gallo et al. 2024), and the intensity at which this accumulated work is completed at (Barranco-Gil et al. 2024; Leo et al. 2022; Mateo-March et al. 2024). Thus, experiments aiming to investigate the physiological concept require the incorporation of a pre-load protocol whilst mimicking the intensities observed in real-life road races. Recent experimental studies have focussed on physiological responses and oxygen uptake kinetics using the exercise intensity domain concept (moderate, heavy, severe and extreme) at intensities where humans transition from one intensity domain into the other, but adopted a fixed time and continuous exercise intensity protocol (Hamilton et al. 2024; Stevenson et al. 2022; Gallo et al. 2024; Brownstein et al. 2022). Emerging evidence suggests that these thresholds play an important role in durability as accumulated work appears to reduce the exercise intensity at which these thresholds occurs with strong associations between the magnitude of this reduction (i.e. fatigue resistance) and performance (Hamilton et al. 2024).

Given the relationship between shifts in exercise intensity domains and durability (Brownstein et al. 2022), it is relevant to assess which strategies of anchoring exercise intensities during the pre-load phase in cycling race simulation protocols have been used. An overview of identified articles following the systematic search, including a breakdown of participant characteristics, environment and equipment, and details on exercise protocols are presented in Table 1. Twelve studies anchored the fractional (%) intensity of the pre-load protocol based on peak power output (PPO) (Palmer et al. 1997; Baume et al. 2008; Christensen et al. 2024; Guillochon and Rowlands 2017; Helge et al. 2023; O'Brien et al. 2023; Rauch et al. 1995; Rauch et al. 1995; Rowlands and Hopkins 2002; Slattery et al. 2014; Stanley et al. 2013; Vaile et al. 2008), 13 based on $\dot{\text{V}}$ O_2max/peak_ (Cureton et al. 2007; Ganio et al. 2011, 2010; Glazier et al. 2004; Goulet et al. 2008; Hargreaves et al. 1984; Hickner et al. 2010; Murray et al. 1991; Paul et al. 2001; Salvador et al. 1985; Sherman et al. 1989; Stebbins et al. 2014; Talanian and Spriet 2016) whilst 13 studies anchored exercise intensity based on physiological variables linked to exercise intensity domains (moderate, heavy, severe, extreme). Specifically, nine studies used lactate outcomes obtained during screening assessments to quantify % exercise intensity, including absolute lactate values (Vandebuerie et al. 1998), maximal lactate steady state (Thienen et al. 2009), traditional lactate thresholds 1 (Poffé et al. 2021a, 1985, 2021b; Robberechts et al. 1985; Dalle et al. 2021) and 2 (Schuylenbergh et al. 2005) or estimation of maximal lactate steady state using D_max_ mathematical modelling (Cramp et al. 2004). Further methods included fractions of ventilatory thresholds (Kremenic et al. 2009; Glace et al. 2019, 2013) and critical power (CP) (Spragg et al. 2023). Other studies-based exercise intensity as a % of 6-min maximal power output (Ørtenblad et al. 2024) used a self-pacing protocol (Burke et al. 1985; Schabort et al. 1998; Hunter et al. 2022; Macdermid et al. 2012; Levin et al. 2014; Abbiss et al. 2010; St Clair Gibson et al. 2001) or used a fixed Watt per kg (W/kg) (Perim et al. 2022) as anchors of exercise intensity.

**Table 1:** Overview of available laboratory-based cycling race simulation protocols, including participant characteristics, equipment and environment of the protocol, pre-load protocol, performance test, inclusion of familiarisation, measurement of reliability, source of justification and usage in other experiements

Reference	Characteristics^a^	Equipment and environment	Pre-load protocol	Performance test	Familiarisation	Reliability	Justification	Used by
Baume et al*.* (Baume et al. 2008)	N = 8 Age: 28.5 ± 4.3 $\dot{\text{V}}$ O_2max_: 63.7 ± 4.5	KingCycle; NR	90 min @ 50% PPO interspersed with 3 × 1 min @ 90% PPO at 10, 35, 80 min and 2 × 4 min @ 70% PPO at 45 and 70 min	20 km TT	Y −1 × 20 km TT	NR	Schabort et al. 1998)	
Christensen et al*.* (Christensen et al. 2024)	N = 18 Age: 28 ± 6 $\dot{\text{V}}$ O_2max_: 71 ± 6	Monark 839E; NR	120 min: 6 × 20 min blocks: 15 min @ 50% PPO followed by 2 × 20 s maximal sprint + 40 s recovery + 2 min recovery	400 kcal TT	Y −1 × Sprints and TT protocol	CV 400 kcal TT: 1.9 ± 1.4% In fresh state	NR	
Cramp et al*.* (Cramp et al. 2004)	N = 8 Age: 22 ± 6.3 $\dot{\text{V}}$ O_2max_: 60 ± 3.7	Lode Excalibur; 21 °C; 30% RH	93 min (4 × 22.5 min laps): Always > 50%D_max_, power alternating every 10 s	6 × 30 s sprints each lap	Y −2x	NR	Palmer et al. 1994) and unpublished power output (SRM) from elite MTB stage	
Cureton et al. (Cureton et al. 2007)	N = 16 Age: 27.5 ± 7, $\dot{\text{V}}$ O_2max_: 60.5 ± 7.2	Lode Excalibur 28.5 °C; 60% RH	120 min alternating every 15 min between 60 and 75% $\dot{\text{V}}$ O_2max_	15-min all-out ride	Y −1x	NR	NR	Ganio et al. 2011; Ganio et al. 2010)
Glazier et al*.* (Glazier et al. 2004)	N = 7 Age: 25 ± 1.1 $\dot{\text{V}}$ O_2max_: 65.3 ± 1.2	Lode Instruments NR	120 min @ 70% $\dot{\text{V}}$ O_2max_ interspersed with 5 × 2 min 85% $\dot{\text{V}}$ O_2max_	7 kJ/kg BM TT	Y −2x	NR	NR	
Goulet et al*.* (Goulet et al. 2008)	N = 6 (1F) Age: 36.5 ± 5.5 $\dot{\text{V}}$ O_2max_: 59.1 ± 4.7	Ergoline ER 900, Jaeger; 27 °C; 55% RH	120 min @ 65% $\dot{\text{V}}$ O_2max_ interspersed by 5 × 2 min @ 80% $\dot{\text{V}}$ O_2max_	Incremental TTE (25W per 3 min)	Pre-load −not TTE	CV of TTE in fresh state 0.9–1.2% based on Hopkins 2001 (Hopkins et al. 2001)	NR	
Guillichon et al*.* (Guillochon and Rowlands 2017)	N = 12 Age: 33.6 ± 9.4 $\dot{\text{V}}$ O_2max_: 66.8 ± 4.7	Velotron RacerMate; 20 °C; 49% RH	140 min. Stochastic between 95 and 37% PPO	Ramp-TTE (0.333 W/s)	Y −1x	CV: 1.3% Ramp-TTE following 150 min continuous cycling based on O'Brien et al*.* 2011 (O'Brien and Rowlands 2011)	Palmer et al. 1997)	
Hargreaves et al. (Hargreaves et al. 1984)	N = 10 Age: 21.8 ± 0.6 $\dot{\text{V}}$ O_2max_: 4.43 ± 0.13 L/min	Collins electrically-braked ergometer NR	240 min (8 × 30 min blocks). 20 min @ 50% $\dot{\text{V}}$ O_2max_, followed by 4 × 30 s @ 100% $\dot{\text{V}}$ O_2max_ + 2 min rest	TTE @ 100% $\dot{\text{V}}$ O_2max_	NR	NR	NR	
Helge et al*.* (Helge et al. 2023)	N = 12 Age: 41.1 ± 6.2, $\dot{\text{V}}$ O_2max_: 61.1 ± 5.0	Monark LC6, 20 °C; 40% RH	150 min @ 60% PPO interspersed at 30 min and 110 min by 15 min incremental steps: 5 min @ 65% PPO, 5 min @ 70% PPO, 5 min 65% PPO	TTE on repeated, incremental intermittent sprint (1 min @ 95%, 100%, 105%, 110% PPO with 2 min recovery @ 65% PPO)	Y −1x	CV: 0.11 in fresh state – unclear if %	NR	
Hickner et al*.* (Hickner et al. 2010)	N = 12 Age: 27.3 ± 1.0 $\dot{\text{V}}$ O_2max_: 53.3 ± 2.0	Lode Diversified, NR	120 min @ 60% $\dot{\text{V}}$ O_2max_ interspersed every 15 min by 2.5 min blocks of 3 × 10 s @ 110% $\dot{\text{V}}$ O_2max_ with recovery @ 65% $\dot{\text{V}}$ O_2max_	Sprint to exhaustion @ 110% $\dot{\text{V}}$ O_2max_	Y −1x, 30 min	NR	NR	
Kremenic et al*.* (Kremenic et al. 2009)	N = 11 Age: 41 ± 3 $\dot{\text{V}}$ O_2max_: 55.7 ± 1.7	Kurt Kinetic Road Machine; NR	120 min @ VT1, interspersed with 5 × 1 min sprints every 20 min	3 km TT	NR	NR	NR	Glace et al. 2019; Glace et al. 2013)
Murray et al*.* (Murray et al. 1991)	N = 10 (2F) Age: 32.5 ± 1.7 $\dot{\text{V}}$ O_2max_: 3.52 ± 0.19 L/min	Velodyne Trainers; 10 °C; 90% RH	120 min. 10 min WU, 50 min @ 65% $\dot{\text{V}}$ O_2max_, 4 × 15 min blocks of 5 min @ 75% $\dot{\text{V}}$ O_2max_ with 10 min @ 65% recovery	4.8 km TT	Y −2x	NR	NR	
O'Brien et al*.* (O'Brien et al. 2023)	N = 19 (4F) Age: 30 ± 9 $\dot{\text{V}}$ O_2max_: 55 ± 8	Velotron RacerMate; NR	90 min @ 65–70% PPO interspersed by 5 × 5 min @ 95% PPO at 15, 35, 45, 65 and 75 min	20 km variable grade TT	Y −1x	CV: 1.1 – 1.5% for 20 km TT in fresh state based on Clark et al*.* 2014 (Clark et al. 2014)	NR	
Ørtenblad et al*.* (Ørtenblad et al. 2024)	N = 12 Age: 23 (21–25), $\dot{\text{V}}$ O_2max_: 73.6 (71.2—76)	Schoberer Rad Messtechnik (SRM), 117 GmbH Julich. NR	240 min. 50% @ MPO 6 min (established in separate visit) interspersed every 30 min by 1 min @ 120% MPO6min and 6-s maximal sprint every 60 min	6-s all-out sprint + 6-min TT	Y −1 × 6 min TT in fresh state	1.1 (0.9) % in fresh state	NR	
Palmer et al*.* (Palmer et al. 1997)	N = 6 Age: 25 ± 8, $\dot{\text{V}}$ O_2max_: 60.2 ± 6.9	KingCycle air-braked ergometer; NR	150 min. stochastic between 35 and 82% PPO, average 58% PPO	20 km TT	NR	NR	Protocol to replicate demands of 105 km outdoor RR (Palmer et al. 1994)	
Paul et al*.* (Paul et al. 2001)	N = 6F, Age: NR $\dot{\text{V}}$ O_2max_: 49.4	Monark; NR	90 min. 6 × 15 min blocks. Each 15 min block was 12 min @ 72% $\dot{\text{V}}$ O_2max_, 1 min @ 100% $\dot{\text{V}}$ O_2max_, 2 min @ 50% $\dot{\text{V}}$ O_2max_	TT: 1500 pedal revolutions as quick as possible	Y −2x	NR	Jeukendrup et al. 1996) but with alternating intensities	
Perim et al*.* (Perim et al. 2022)	N = 17 Age: 38 ± 9 $\dot{\text{V}}$ O_2max_: 52.4 ± 8.3	Lode Excalibur, NR	120 min. 10-min blocks of variable intensity (2 × 1.5 w/kg BM, 6 × 2.0 w/kg, 2 × 2.5 w/kg, 2 × 3.0 w/kg). 10-s max sprints every 20 min	4 km time-trial @ 5% gradient	Y −2x	NR	Sanders and Heijboer 2019; Vogt et al. 2006)	
Poffe et al*.* (Poffé et al. 1985)	N = 12 Age: 25 ± 6, $\dot{\text{V}}$ O_2max_: 62.4 ± 6.6	Avantronic Cyclus 2; 18 °C; 60% RH	180-min. 6 × 30 min blocks, each block was divided in 5-min intervals of 60, 70, 90, 70, 80 and 60% of LT1	15-min TT and TTE @ 175% LT—5 min rest between	Y −2x	NR	NR	Poffé et al. 2021a; Poffé et al. 2021b; Robberechts et al. 1985) (Dalle et al. 2021)
Rauch et al*.* (Rauch et al. 1995)	N = 8 Age: 22.4 ± 0.6 $\dot{\text{V}}$ O_2max_: 66.3 ± 1.3	KingCycle air-braked ergometer; NR	120 min @ 65% PPO interspersed by 5 × 1 min @ 100% PPO every 20 min	1 h TT	Equipment familiarisation	NR	Palmer et al. 1994)	Rauch et al. 1995)
Rowlands et al*.* (Rowlands and Hopkins 2002)	N = 12 Age: 27 ± 8 $\dot{\text{V}}$ O_2max_ 64 ± 6	KingCycle air-braked ergometer; 19–21 °C; 45–55% RH	110 min. 60 min @ 50% PPO followed by 10 min stages @ 55%, 65%, 70%, 75% and 82% PPO	50 km TT, including 1 km and 4 km All out	Yes 1x	Based on (Schabort et al. 1998)	Schabort et al. 1998)	
Salvador et al*.* (Salvador et al. 1985)	N = 12 (3F) Age: 31 ± 9 $\dot{\text{V}}$ O_2max_: 60.7 ± 9.0	Lode Excalibur; NR	120 min @ 60% $\dot{\text{V}}$ O_2peak_ interspersed every 30 min by 4 × 3 min @ 85% $\dot{\text{V}}$ O_2peax_	TT 6 kJ/kg BM	Y −1x	NR	NR	
Schabort et al*.* (Schabort et al. 1998)	N = 8 Age: 26 ± 3.5, $\dot{\text{V}}$ O_2max_: 64.8 ± 5.7	KingCycle air-braked ergometer; NR	100 km distance self-paced TT with 4 × 1 km all-out (10 km, 32 km, 52 km, 72 km) and 4 × 4 km all-out (20 km, 40 km, 60 km, 80 km)	NR^*^	Y – 1x	CV time 100-km: 1.7% CV average time 1 km: 1.9% CV average time 4 km 2.0%	Palmer et al. 1994)	Burke et al. 1985; Hunter et al. 2002; Macdermid et al. 2012; Levin et al. 2014; Abbiss et al. 2010; St Clair Gibson et al. 2001)
Sherman et al*.* (Sherman et al. 1989)	N = 10 (2F) Age: 30 ± 3 $\dot{\text{V}}$ O_2max_: 4.0 ± 0.2 L/min	Monark; NR	95 min. 5 × 15 min @ 70% $\dot{\text{V}}$ O_2max_ interspersed with 4 × 5 min @ 52% $\dot{\text{V}}$ O_2max_	TT—complete amount of revolutions as quickly as possible—equivalent to 45 min @ 70% $\dot{\text{V}}$ O_2max_	Y −1x	NR	NR	
Spragg et al*.* (Spragg et al. 2023)	N = 10 Age: 19.2 ± 0.8 $\dot{\text{V}}$ O_2max_: 74.4 ± 4.8	Cyclus2; 19–22 °C; 40–50% RH	140 min: 20 min @ 50–70% CP, 5 × 8 min @ 105–110% CP with 8 min recovery between (RPE < 2/10). Followed by 40 min active recovery (RPE < 2/10)	3 min and 12 min CP test, separated by 40 min active recovery (RPE < 2/10)	NR	NR	NR	
Stebbins et al*.* (Stebbins et al. 2014)	N = 8 Age: 35 ± 2 $\dot{\text{V}}$ O_2max_: 64.0 ± 4.3	Velotron RacerMate; NR	190 min: 30 min @ 65% $\dot{\text{V}}$ O_2max_ followed by 4 × 40 min blocks: 12 min @ 80% $\dot{\text{V}}$ O_2max_, 8 min @ 65% $\dot{\text{V}}$ O_2max_, 10 min @ 50% $\dot{\text{V}}$ O_2max_, 10 min @ 65% $\dot{\text{V}}$ O_2max_	Ramp-TTE (25 W/min)	NR	NR	HR response from single and multi-day race (Palmer et al. 1994; Luciá et al. 1999)	
Talanian et al*.* (Talanian and Spriet 2016)	N = 15 (4F) Age: 22.5 ± 0.9 $\dot{\text{V}}$ O_2max_: 64.6 ± 1.9	Lode excalibur; 19–22 °C; 20–30% RH	120 min @ 60% $\dot{\text{V}}$ O_2peak_ interspersed every 20 min by 5 × 2 min @ 82% $\dot{\text{V}}$ O_2peak_ with 40 s @ 50W recovery	6 kJ/kg BM	Y −1x	CV: 2.4%	NR	
Vaile et al*.* (Vaile et al. 2008)	N = 12 Age: 32.2 ± 4.3 $\dot{\text{V}}$ O_2max_: 68.8 ± 3.6	Kurt Kinetic Road Machine; NR	105 min. 10 min WU, 3 blocks of sprints. Block 1: 36 × 5 s sprint, block 2: 18 × 10 s sprint, block 3: 12 × 15 s sprint. Each block separated in 3 sets with work:rest ratio of 1:6, 1:3, 1:1. Recovery was at 40–50% PPO. Each block separated by 10 min active recovery + 2 min TT. After final block, 5 min active recovery followed by 5 min TT and 5 min active recovery	9 min Total TT; 5 min TT end	Y −3x	Unpublished, self-reported typical error of 2.1%—unclear to what measurement	NR	Slattery et al. 2014; Stanley et al. 2013)
Van Schuylenbergh et al*.* (Schuylenbergh et al. 2005)	N = 9 Age: 25.6 ± 1.9 $\dot{\text{V}}$ O_2max_: 65.9 ± 2.7	Avantronic Cyclus 2, 20 °C; 60% RH	~ 170 min: 20 min WU—ramp test (~ 20 min)- 2 h intermittent. Intermittent was 2 cycles of 5 min 65% LT2, 2 × 10 min @ 85% LT2 and 3 × 5 min @ 100% LT2. 5 min Recovery between blocks @ 65% LT2	TTE—Ramp test (5 W every 30 s)	NR	NR	NR	
Van Thienen et al*.* (Thienen et al. 2009)	N = 19 Age: 24.9 (18–30) $\dot{\text{V}}$ O_2max_: 60.3 (45–72)	Avantronic Cyclus 2; 18 °C; 60% RH	110 min, 10 min blocks of variable intensity (1 × 50% MLSS, 1 × 60% MLSS, 4 × 70% MLSS, 2 × 80% MLSS, 2 × 90% MLSS)	10 min TT. 30 s sprint	Y −1x	Self-reported for TT on n = 7: ICC 0.97, unclear if fresh or following race simulation	NR	
VandeBueri et al*.* (Vandebuerie et al. 1998)	N = 12 Age: 23 (21–25) $\dot{\text{V}}$ O_2peak_: 73.6 (71.2—76)	Technogym Spintrainer; NR	150 min. 30 min @ 1 mmol/L, 45 min @ 2 mmol/L, 5 × 5 min @ 3 mmol (5 × 5 min recovery @ 1 mmol), 20 min 1 mmol/L, 5 min 2 mmol/L 5 min 3 mmol/L	TTE @ 4 mmol/L + 5 × 10 s max sprint with 2 min rest	NR	NR	NR	

^a^Male participants unless stated otherwise (F)

^*^Original protocol does not have a final 1 km TT performance, whereas Burke et al*.* 2000 and Hunter et al*.* 2002 include a final 1 km TT

*BM* Body mass, *CP* Critical power, *CV* Coefficient of variation, *ICC* Intra-class correlation, *LT* Lactate threshold, *MPO*_*6min*_ Mean power output in 6 min TT, *MLSS* Maximum lactate steady state, *NR* Not reported, *PPO* Peak power output, *RH* Relative humidity, *RPE* Rate of perceived exertion, *TT* Time-trial, *TTE* Time-to-exhaustion, *WU* Warming-up

When translating the above methods into the reality of road races and the construct of fatigue resistance, each methods has strengths and limitations (Table 2). The majority of pre-load protocols used %PPO or $\dot{\text{V}}$ O_2max/peak_ as exercise intensity anchor. These methods have shortcomings which are critiqued elsewhere (Jamnick et al. 2020). Instead, using exercise intensity domains (LT_1_/VT_1_ and/or VT_2_/LT_2_/CP) from the perspective of establishing mechanisms of fatigue resistance is worthwhile as there is evidence that working above or below such thresholds differentially influence performance when matched for total work (Leo et al. 2022; Mateo-March et al. 2024), most likely due to a different metabolic environment and perturbations (Jamnick et al. 2020; Iannetta et al. 2020). It is currently unclear what the exact underlying reasons for this are, but some evidence points towards utilisation of different muscle glycogen pools when exercising at different intensities and carbohydrate availability (Clark et al. 1985; Jensen et al. 2020a; Schytz et al. 2024). Subsequently, glycogen depletion is linked to reduced skeletal muscle contractile function which might be a consequence of impaired sodium–potassium ATP-ase (Na + -K + -ATPase) enzyme activity (Cairns and Renaud 2023; Jensen et al. 2020b). In the current search, protocols that used exercise domains as anchors, limitations exist. Some protocols had their intensity below LT_1_ for the full pre-load (Poffé et al. 2021a, 1985, 2021b; Robberechts et al. 1985; Dalle et al. 2021), which might not be representative of cycling race intensity distribution (Sanders and Heijboer 2019). Others used the boundary between the heavy and severe domain (LT_2_, CP or maximum lactate steady state) as anchor, but it was unclear whether intensities ever dropped into the moderate domain (Thienen et al. 2009; Schuylenbergh et al. 2005; Spragg et al. 2023).

**Table 2:** Overview of strengths, limitations and recommendations when selecting a method for anchoring exercise intensities during a pre-load protocol to induce fatigue

	Strengths	Limitations	Recommendation
Exercise intensity domains	• Control for metabolic perturbations • Relative to training status	• Does not match for total work in a fixed-time protocol in a heterogeneous fitness status sample • Real-life road races do not follow fixed patterns/blocks (stochastic) • Validity of measurement protocols can be poor	• Use when objective is to establish physiological mechanisms • Avoid fixed-time protocol, match for work to account for variability in training status
%$\dot{\text{V}}$ O_2max/peak_ HR or PPO,	• Relative to individuals’ maximal capacity • Simple to measure and calculate	• Does not match for total work in a fixed-time protocol • Does not align with exercise intensity thresholds in a heterogeneous fitness sample	• Not recommended for use. Use exercise intensity domains instead
Fixed distance, self-selected power	• Aligns with real-world scenarios • Can closely match for work (kJ or kJ/kg BM) • Potential to use computer software to mimic real racing scenarios	• Most options do not account for air resistance (e.g. drafting) or frontal area experienced during outdoor road races • Difficult to replicate • Variability in work spend in different exercise intensity domains	• Include ‘intervals’ to introduce real-life scenarios (attacks, hills etc.) • Analysis of accumulated work in domain required • Potential to further develop with computer software
Fixed work (kJ or kJ/kg BM)	• Homogeneity in fatigue • Reduces potential effects of pacing and drafting in racing	• Absolute values do not discern between different sizes of riders • Considerations required for how much work untrained individuals can perform	• Use of relative amount of work (kJ/kg BM). Recommended especially for efforts shorter than 20 min (i.e. severe exercise intensity domain) • When combined with exercise intensity domain, match for work in each domain

*BM* Body mass, *HR* Heart rate, *kJ* Kilojoule, *PPO* Peak power output, $\dot{V}$ *O*_*2max*_ Maximal oxygen uptake capacity

In addition to the limitations raised above, when designing a pre-load protocol to induce fatigue prior to a performance test, heterogeneity in the fitness or training status and body composition between participants need to be considered. Differences in these variables mean that the absolute and relative power outputs at exercise domain thresholds (LT) are inconsistent between individuals (Joyner and Coyle 2008). Subsequently, when using a fixed-time protocol, the accumulated work before the performance test will vary between participants as well. Again, this could be problematic as fatigue resistance in the field is analysed based on kJ, rather than time (Erp et al. 2021). As an example: in Hamilton et al*.* (Hamilton et al. 2024) participants cycled for 150 min at 90% of VT_1_. Inspecting individual data points (Fig. 3a), the lowest constant-power output was ~ 125W and the highest was ~ 250W. This means that the individual with the higher power output accumulated double the amount of absolute work after 150 min (1125 kJ vs. 2250 kJ). First, we propose that future experiments present and normalise the work relative to body mass since larger individuals will likely produce higher power. Second, if these two individuals had similar body mass and adopt a matched-work protocol, the high would only have to exercise for half the time, therefore likely experiencing less fatigue. Thus, to overcome these issues, we propose future race simulation protocols to match their pre-load for accumulated work relative to body weight in each exercise domain (e.g. spend 10 kJ/kg BM in moderate domain, 5 kJ/kg BM in heavy domain and 2 kJ/kg BM in severe domain).

When further translating pre-load protocols to road racing conditions it should be noted that in real-life, all cyclists are required to cover a set distance rather than a set amount of time. Although over flat terrain cyclists often arrive at the same time due to the enhanced effect of drafting in the peloton, races over mountainous terrains have reduced draught benefits and rely more on power output relative to body mass (W/kg). To account for such scenarios, a fixed-distance (or amount of work) protocol might be a preferred, where self-selected intensity interspersed with high-intensity efforts to simulate ‘attacks’ or ‘climb’ provide a more realistic protocol as has been used by several papers (Burke et al. 1985; Schabort et al. 1998; Hunter et al. 2002; Macdermid et al. 2012; Levin et al. 2014; Abbiss et al. 2010; St Clair Gibson et al. 2001).

To support further development of variable-intensity race simulation protocols, it is worthwhile to inspect observational data from road races. Power outputs produced by elite athletes as external load are often not attainable by less trained cyclists. However, the recording of internal load e.g. time spent at % of maximal heart rate (HR_max/peak_) could provide another alternative for prescribing exercise intensities for a pre-load protocol. For example, Gallo and colleagues reported both external and internal load in junior, under-23 and professional category races over a full season and reported that in the professional races roughly ~ 15%, ~ 25%, ~ 30%, ~ 20% and ~ 5% of time in a race was spent at 50–59, 60–69, 70–79, 80–89 and 90–100% HR_peak_ respectively (Gallo et al. 2022). However, fraction of HR_max_ comes with the similar limitations as %$\dot{\text{V}}$ O_2max_ where % at which an individual crosses into a different exercise domain is dependent on training status. Thus, Sanders & Heijboer (Sanders and Heijboer 2019) provided power and heart-rate distributions using the three-zone exercise domain (zone 1: < LT_1_, zone 2: > LT_1_, < LT_2_ and zone 3, > LT_2_) for various types of road races during a grand tour stage race, demonstrating that for example in a mountain stage ~ 70% of time was spent in zone 1, ~ 10% in zone 2 and ~ 20% in zone 3. Such observational data could be used to design pre-load protocols, which has been mentioned as justification only by Perim et al. (Perim et al. 2022) however in their experiment, the highest power output during the pre-load was 3.0 W/kg (214 ± 26 W), with no blood lactate data to identify exercise intensity domains but which for elite or even trained cyclist would be < LT_1_.

It should be noted that during cycling road races, due to strategic decisions and effects of terrain, an athlete has no full authority over selecting their own exercise intensity to be within an intensity domain and merely needs to produce the power not to get dropped. Lastly, fatigue resistance induces changes in physiological parameters, such as an increase in heart rate(Stevenson et al. 2022; Deaner et al. 2015), reductions in CP, (Clark et al. 2018) and lactate threshold (Stevenson et al. 2022). These factors are currently not considered when designing pre-load protocols.

The types of performance test following the pre-load were time-to-exhaustion (Guillochon and Rowlands 2017; Helge et al. 2023; Goulet et al. 2008; Hargreaves et al. 1984; Hickner et al. 2010; Stebbins et al. 2014; Schuylenbergh et al. 2005), time trials with fixed amount of work to be performed (i.e. distance) (Palmer et al. 1997; Baume et al. 2008; O'Brien et al. 2023; Rowlands and Hopkins 2002; Murray et al. 1991; Kremenic et al. 2009; Glace et al. 2019, 2013; Burke et al. 1985; Schabort et al. 1998; Hunter et al. 2002; Macdermid et al. 2012; Levin et al. 2014; Abbiss et al. 2010; St Clair Gibson et al. 2001; Perim et al. 2022), work (Christensen et al. 2024; Glazier et al. 2004; Paul et al. 2001; Salvador et al. 1985; Sherman et al. 1989; Talanian and Spriet 2016) or time (Rauch et al. 1995, 2005; Slattery et al. 2014; Stanley et al. 2013; Vaile et al. 2008; Cureton et al. 2007; Ganio et al. 2011, 2010), maximal sprints (Cramp et al. 2004), multiple fixed time all-out tests for CP determination (i.e. 3 min and 12 min) (Spragg et al. 2023) or a combination of multiple performance tests (Vandebuerie et al. 1998; Thienen et al. 2009; Poffé et al. 2021a, 1985, 2021b; Robberechts et al. 1985; Dalle et al. 2021; Ørtenblad et al. 2024). Clearly on many occasions, the winner of a road race is decided by a final effort, hence this review evaluated the type of performance tests used in the simulations. The type of final effort is mostly dictated by the demands of the terrain. For example, flat road races that often finish in a ‘mass sprint’ are characterised by gradual increase in power output with repeated short power surges (positioning) followed by a final maximal sprint (Menaspà et al. 2015). Mountain stages with an uphill finish often follows a pattern of graded increases in power output, followed by a short, high-intensity effort (‘attack’), followed by a ‘time-trial’ to the finish line. None of the scoped articles followed such intensity patterns within their performance test and therefore provided limited translatability to the real world. Articles that contained multiple performance tests at the end often included a break or phase of low-intensity recovery. If the primary outcome of an experiment is performance-based instead of physiology-based, it is worth to establish new performance tests that mimic these scenarios more closely.

## Examination of nutritional strategies

Table 3 contains scoring of nutrition protocols used in the included studies. Of the 30 unique protocols, overall, ten studies had an adequate nutritional protocol implemented (Guillochon and Rowlands 2017; Rauch et al. 1995; Murray et al. 1991; Paul et al. 2001; Salvador et al. 1985; Vandebuerie et al. 1998; Thienen et al. 2009; Poffé et al. 1985; Schuylenbergh et al. 2005; Cramp et al. 2004), 15 studies followed a limited nutritional protocol (Palmer et al. 1997; Baume et al. 2008; Helge et al. 2023; O'Brien et al. 2023; Rowlands and Hopkins 2002; Cureton et al. 2007; Glazier et al. 2004; Goulet et al. 2008; Hickner et al. 2010; Sherman et al. 1989; Talanian and Spriet 2016; Kremenic et al. 2009; Ørtenblad et al. 2024; Schabort et al. 1998; Perim et al. 2022) and five studies had an inadequate control over nutritional intake (Christensen et al. 2024; Vaile et al. 2008; Hargreaves et al. 1984; Stebbins et al. 2014). The most insufficiently controlled phase was nutrition in the day before the experimental trial with an average score of −0.6 ± 1.2 (Possible score range −2– + 2). Six studies provided athletes with adequate nutrition guidelines and/or actual foods that would achieve high muscle and liver glycogen stores on the day of the trial (Guillochon and Rowlands 2017; Rauch et al. 1995; Paul et al. 2001; Salvador et al. 1985; Poffé et al. 1985; Cramp et al. 2004). Nutrition in the hours before the trial was on average scored as −0.1 ± 1.7, with 14 studies sufficiently controlling nutrition prior the trial (Baume et al. 2008; Guillochon and Rowlands 2017; Rauch et al. 1995; Rowlands and Hopkins 2002; Goulet et al. 2008; Murray et al. 1991; Paul et al. 2001; Salvador et al. 1985; Sherman et al. 1989; Vandebuerie et al. 1998; Thienen et al. 2009; Poffé et al. 1985; Schuylenbergh et al. 2005; Cramp et al. 2004). The phase with the most adequate nutritional control was during the trials (mean score 1.1 ± 1.3) where 23 studies sufficiently controlled the nutrition.

**Table 3:** Nutritional intake protocols and scoring indexes

		Day(s) before trial		Hours before trial		During trial		
Reference	Duration of exercise	Details	Score	Details	Score	Details	Score	Total
Baume et al*.* (Baume et al. 2008)	90 min + ~ 30 min	Self-reported diet log for 24 h period before; a small dinner meal (i.e. 56 g CHO) was provided Total CHO not reported	−1	A standardised meal was provided before the trial (45 gr CHO) Not in line with recommendations	+ 1	CHO provided at a rate of 60 gr/h CHO In line with the recommendation	+ 2	+ 2
Christensen et al*.* (Christensen et al. 2024)	120 min + ~ 20 min	Participants asked to standardise the nutrition. No quantities reported	−1	Participants asked to standardise the nutrition. No information provided on quantity	−1	No information provided	−2	−4
Cramp et al*.* (Cramp et al. 2004)	93 min	Dietary food log provided and analysed (~ 6.5 gr CHO/kg BM) In line with recommendations	+ 2	Meals provided (3 g CHO/kg BM) 3 h pre-exercise In line with recommendation	+ 2	Water provided Not in line with recommendations	0	+ 4
Cureton et al*.* (Cureton et al. 2007)	120 min + 15 min	Received instructions to consume mixed diet for 2 days before the trial No quantities reported	−1	Fasted or 3 h postprandially. No information on quantity	−1	CHO provided at rate of ~ 72 gr/h In line with recommendations	+ 2	0
Glazier et al. (Glazier et al. 2004)	120 min + ~ 35 min	Dietary food log 24 h before. No quantities reported	−1	No information provided	−2	CHO provided at rate of ~ 66 gr/h In line with recommendations	+ 2	−1
Goulet et al*.* (Goulet et al. 2008)	120 min + ~ 15 min	Dietary food log for 24 h before. No quantities reported	−1	Food item provided 1 h pre-exercise (white bagel, 120 kcal) Not in line with recommendations	+ 1	CHO provided at rate of ~ 66 gr/h In line with recommendations	+ 2	+ 1
Guillichon et al*.* (Guillochon and Rowlands 2017)	140 min + ~ 10 min	Food provided (5.5 g/kg BM CHO) Not in line with recommendations	+ 1	A standardised lunch was provided at 3 h and 1 h pre-exercise (> 1.5 gr CHO/kg BM) In line with recommendations	+ 2	CHO provided at a rate of 80 gr/h In line with recommendations	+ 2	+ 5
Hargreaves et al*.* (Hargreaves et al. 1984)	240 min + ~ 2 min	No information provided	−2	No information provided	−2	CHO provided at a rate of ~ 43 gr/h Not in line with recommendations	+ 1	−3
Helge et al. (Helge et al. 2023)	150 min + ~ 15 min	No information provided	−2	No information provided	−2	CHO provided at 80 g in total (32 gr/h) In line with recommendations	+ 2	−2
Hickner et al*.* (Hickner et al. 2010)	120 min + ~ 1 min	Dietary food log 24 h before. No quantities reported	−1	Diet record but no information on quantities	−1	Water ad libitum Not in line with recommendations	0	−2
Kremenic et al*.* (Kremenic et al. 2009)	120 min + ~ 4 min	Instructed “*to eat as they would normally do before racing”*	−1	No information provided	−2	Water provided Not in line with recommendations	0	−2
Murray et al*.* (Murray et al. 1991)	120 min + ~ 8 min	Instructed to standardise nutrition 24 h before. No quantities reported	−1	Breakfast and lunch provided. ~ 2 gr CHO/kg BM In line with recommendations	+ 2	CHO provided at rates of 78 gr/h In line with recommendations	+ 2	+ 3
O'Brien et al*.* (O'Brien et al. 2023)	90 min + ~ 45 min	No information provided	−2	Fasted Not in line with recommendations	−1	CHO provided at a rate of ~ 66gr/h In line with recommendations	+ 2	−1
Ørtenblad et al*.* (Ørtenblad et al. 2024)	240 min + ~ 6 min	Instructed “*to treat the nutritional preparation as they would before competition”.* No information on quantity provided	−1	Instructed “*to treat the nutritional preparation as they would before competition”.* No information on quantity provided	−1	CHO provided at a rate of 100 gr/h In line with recommendations	+ 2	0
Palmer et al*.* (Palmer et al. 1997)	150 min + ~ 25 min	Dietary control but no quantities provided	−1	Diet record but no information on quantities	−1	CHO provided at a rate of 40 gr/h Not in line with recommendations	+ 1	−1
Paul et al*.* (Paul et al. 2001)	90 min + ~ 12 min	Food provided (10 gr/kg BM CHO 48 h before trial) In line with recommendations	+ 2	Breakfast and lunch provided (CHO intake 8 gr/kg BM) In line with recommendations	+ 2	Water provided ad libitum Not in line with recommendations	0	+ 4
Perim et al*.* (Perim et al. 2022)	120 min + ~ 12 min	Dietary food log 24 h before. No quantities reported	−1	Dietary food log morning. No quantities reported	−1	CHO provided at a rate of 36 gr/h In line with recommendations	+ 2	0
Poffe et al*.* (Poffé et al. 1985)	180 min + ~ 20 min	Dinner provided ~ 3 gr/kg BM CHO but no information provided on total daily intake	+ 1	Breakfast provided (~ 1.5 gr CHO/kg BM 90 min pre-exercise In line with recommendations	+ 2	CHO provided at a rate of 60 gr/h In line with recommendations	+ 2	+ 5
Rauch et al*.* (Rauch et al. 1995)	120 min + 60 min	Supplements provided for 3 days to meet intake of 8–12 gr/kg BM CHO. Dietary food log 24 h before. No quantities reported In line with recommendations	+ 2	Standardised breakfast provided. ~ 75 gr CHO 3 h pre-exercise In line with the recommendations	+ 2	CHO provided at a rate of ~ 60 gr/h Not in line with recommendations	+ 2	+ 6
Rowlands et al*.* (Rowlands and Hopkins 2002)	110 min + ~ 70 min	Dietary food log 24 h before. No quantities reported	−1	Standardised meal provided 90 min pre-exercise (~ 3.4 gr CHO/kg BM) In line with the recommendations	+ 2	CHO provided at a rate of ~ 55 gr/h Not in line with recommendations	+ 1	+ 2
Salvador et al*.* (Salvador et al. 1985)	120 min + ~ 10 min	Meals provided (7 gr/kg BM CHO) In line with recommendations	+ 2	Standardised breakfast provided 2 h pre-exercise (1 gr CHO/kg BM) Not in line with recommendations	+ 2	CHO provided at a rate of 60 gr/h In line with recommendations	+ 2	+ 6
Schabort et al*.* (Schabort et al. 1998)	~ 150 min	Dietary food log 24 h before. No quantities reported	−1	Diet record but no information on quantities	−1	CHO provided ad libitum. Amounts replicated on subsequent trials No information on quantities provided	0	−2
Sherman et al*.* (Sherman et al. 1989)	95 min + ~ 45 min	Menu provided and advice given. No information on quantities	−1	Meals provided 4-h pre-exercise (~ 2 or ~ 4 gr CHO/kg BM) In line with recommendations	+ 2	No information provided	−2	−1
Spragg et al*.* (Spragg et al. 2023)	140 min + 55 min	No information provided	−2	No information provided	−2	CHO provided at a rate of 60 gr/h In line with recommendations	+ 2	−3
Stebbins et al*.* (Stebbins et al. 2014)	180 min + ~ 10 min	No information provided	−2	No information provided	−2	CHO provided at a rate of 40 gr/h Not in line with recommendations	+ 1	−3
Talanian et al*.* (Talanian and Spriet 2016)	120 min + ~ 26 min	Dietary pre-race routine followed. No quantities reported	−1	Dietary pre-race routine followed. No quantities reported	−1	CHO provided at a rate of 60 gr/h In line with recommendations	+ 2	0
Vaile et al*.* (Vaile et al. 2008)	105 min	Dietary food log 24 h before. No quantities reported	−1	No information provided	−2	No information provided	−2	−5
Van Schuylenbergh et al. (Schuylenbergh et al. 2005)	~ 170 min + ~ 10 min	Dietary food log, instructed to follow a CHO-rich diet and provided with ~ 150 gr CHO (2 gr/kg BM). No total daily quantity stated	−1	Standardised breakfast provided 2 h pre-exercise (~ 330 gr CHO or ~ 4 gr CHO/kg BM) In line with recommendations	+ 2	CHO provided at a rate of ~ 75 gr/h In line with recommendations	+ 2	+ 3
Van Thienen et al*.* (Thienen et al. 2009)	110 min + ~ 10 min	Standardised dinner provided (228 gr CHO or ~ 3.1 gr/kg BM). No overall daily quantity provided	−1	Standardised breakfast provided 2 h pre-exercise (240 gr CHO or ~ 3.3 gr CHO/kg BM) In line with recommendations	+ 2	CHO provided at a rate of ~ 70 gr/h In line with recommendations	+ 2	+ 3
Vandebuerie et al*.* (Vandebuerie et al. 1998)	150 min + ~ 25 min	Dietary food log 24 h before. No quantities reported	−1	Standardised breakfast provided 2–3 h pre-exercise (~ 134 gr CHO or ~ 1.8 gr /kg BM) In line with recommendations	+ 2	CHO provided at a rate of 60 gr/h In line with recommendations	+ 2	+ 3

Strength of dietary control in relation to carbohydrate (CHO) intake using an adapted scoring framework from Close and colleagues (Close et al. 2019). Scores (−1, 0, + 1) were assigned to qualitative and quantitative statements on dietary provision. For qualitative evaluation, −1 was assigned when no statement was present whether dietary intake was controlled, 0 was assigned if there was a statement on dietary control, but based on self-reported adherence or where advice-only was given. + 1 was assigned if dietary control was present with participants receiving food products from the research team. For quantitative scores, −1 was assigned if no information on quantities was provided, 0 where quantities are stated but not in line with guidelines and + 1 if quantities were stated and in line with guidelines. For studies that investigated different quantities of CHO either before or during exercise, the highest quantity is recorded

Nutrition and especially carbohydrate intake undoubtedly plays a crucial role in prolonged endurance events (Podlogar and Wallis 2022) and has been shown to directly affect time to task failure and/or performance with nutrition in days leading to the exercise bout (Hawley et al. 1997; Bergström et al. 1967), on the day of the bout (Aird et al. 2018) and during the exercise bout (Stellingwerff and Cox 2014). Therefore, adequate nutritional control regarding carbohydrate intake is required to minimise variability in performance due to fatigue derived from low carbohydrate availability, unless the experimental objective is to directly assess the impact of carbohydrate availability on fatigue resistance. For instance, fatigue resistance has been shown to be affected by carbohydrate intake during exercise (Clark et al. 1985). Whilst the mechanisms for their action remain unclear, it has been hypothesised that the rate of utilisation of endogenous carbohydrate stores could affect fatigue resistance (Spragg et al. 2023) and whilst there is direct evidence for muscle function to be impaired with reduction of muscle glycogen (Nielsen et al. 2024; Ørtenblad et al. 2013), majority of studies do not see skeletal muscle sparing to occur with varying carbohydrate ingestion rates (Areta and Hopkins 2018). On the other hand, liver glycogen depletion is heavily affected by carbohydrate ingestion rates (Jeukendrup et al. 1999; Gonzalez et al. 2016). Whilst the importance of carbohydrates for fatigue resistance are very clear and well established, further research is required to better understand the mechanisms behind these improvements. It is thus very important that nutrition in studies is strictly controlled and either constant or well manipulated so that the findings of studies are not affected by lack of nutrition control. Unfortunately, most studies in this review did have inadequate or limited control on carbohydrate and dietary intake, particularly before exercise, and thus introduced an important results’ confounder. It is of paramount importance that studies sufficiently standardise the nutrition in the trials and match it with current guidelines, so that the potential mechanistic or performance changes found in the studies, are not influenced by insufficient nutrition strategies. In addition to this, standardising nutrition to the highest possible degree might improve reliability of the protocols.

## Reliability and justification of the simulation protocol

Of the thirty unique protocols, nineteen studies did not demonstrate or provide data on reliability of the performance test when completed after the pre-load e.g., in a fatigued state. Four studies justified the reliability of their performance test based on previous studies (Guillochon and Rowlands 2017; O'Brien et al. 2023; Rowlands and Hopkins 2002; Goulet et al. 2008), although those previous studies obtained reliability data in the fresh state (Clark et al. 2014; Hopkins et al. 2001), after a fixed-intensity exercise pre-load (O'Brien and Rowlands 2011) or completed half the distance (Rowlands and Hopkins 2002; Schabort et al. 1998). One study measured reliability of average repeated efforts and overall completion time (Schabort et al. 1998), but not for the final performance test. Two studies reported reliability outcomes from unpublished data within their lab, but it was unclear whether the data were obtained in the fresh or fatigued state (Vaile et al. 2008; Thienen et al. 2009). Three studies provided measurements of reliability within their study sample, but outcomes were obtained in a fresh, non-fatigued state (Christensen et al. 2024; Helge et al. 2023; Ørtenblad et al. 2024). Only one study completed a test–retest of the performance test within the study sample following the pre-load protocol (Talanian and Spriet 2016).

Reliability of outcome measurements is a cornerstone in scientific research. Ideally, measurements of reliability are obtained in a relevant setting. In the case of our research question this means in a fatigued state. Worryingly, only one study provided reliability data for such measurements (Talanian and Spriet 2016). In the past, literature has provided overviews of the reliability of several performance tests, most of which in the fresh state (Currell and Jeukendrup 2008; Hopkins et al. 2001). There are some studies that have measured reliability of performance following a pre-load using cycling exercise, however these studies used a fixed-intensity and the pre-load was shorter than 90 min, therefore not meeting our inclusion criteria (Jeukendrup et al. 1996; Sewell and McGregor 2008; Doyle and Martinez 1998; Currell et al. 2006). For a few reasons, we propose that these protocols of tests are limited and require more research into reliability. First, it is common that cycling road races have an extended duration compared to the fixed-intensity pre-load tests. With increased accumulation of work, fatigue resistance appears to become more prominent in reducing performance (Erp et al. 2021), thus in an experimental sample it could be expected that both within (Hopkins et al. 2001) and between-subject CV (Erp et al. 2021) might increase. Second, variable or stochastic intensity exercise or distribution of exercise intensity across the several exercise domains differentially affects performance (Leo et al. 2022; Mateo-March et al. 2024). It is conceivable that this could have a knock-on effect within cyclist on the reliability in their performance. Herein, it is suggested that performances following pre-load display larger CV than shorter exercise performance tests (Hopkins et al. 2001). This might be a result of day-to-day variability in fatigue resistance, a parameter which is also currently unknown, in the individuals partaking in the experiments. Thus, given that only one study in our review tested reliability of performance following a variable-intensity pre-load, this area warrants more attention in the future studies that aim to address questions pertaining fatigue resistance following variable-intensity exercise. It needs to be acknowledged that the CV in this study was acceptable (2.4% CV) (Talanian and Spriet 2016).

Of the thirty unique protocols, twenty did not provide scientific justification for the pre-load protocol, although several papers stated that the pre-load contained high-intensity efforts “to simulate hill climbs” or create break-aways. Some studies (Palmer et al. 1997; Rauch et al. 1995; Stebbins et al. 2014; Schabort et al. 1998) developed the pre-load protocol based on the heart rate profiles from a road-race (Palmer et al. 1994), which others then used for their protocol (Baume et al. 2008; Guillochon and Rowlands 2017; Rowlands and Hopkins 2002; Cramp et al. 2004). One study (Perim et al. 2022) used more recent field-data, including power readings (Sanders and Heijboer 2019; Vogt et al. 2006) to justify their protocol. Whilst some studies attempted to provide justification for the protocols used, most studies do not report reasons for selection of the pre-load and/or performance protocol. Whilst several papers did provide justification based on real-world data, this data was derived from heart rate distribution of a single race of several decades ago, which might not reflect current racing demands. With the introduction of power meters, more insight into power zone distribution can be obtained and used to design laboratory-based cycling race protocols. Some studies identified by this review have used such observational data, but we encourage researchers to utilise these sources and consider this during protocol development.

## Future directions and recommendations

This review has created a list of available laboratory-based cycling protocols that aimed or claimed to be in line with the physical demands of real-world road-cycling races. One of our aims was that this overview of available protocols serves as a useful starting point for future researchers to design protocols that are closely in line with the demands of cycling road races, for example by optimising the distribution of exercise intensities spent in different exercise domains or by making the performance test more closely aligned with real-life cycling races as outlined above. With the wealth of observational data from the professional field over the recent years, protocols can and should be improved. However, it is prudent that before protocols are being implemented, measurements of reliability are included. Following the establishment of reliable and ecologically valid protocols, these can be used to induce appropriate levels of fatigue which could then subsequently be used to investigate mechanisms of fatigue resistance and interventions to improve. One important note is the underrepresentation of female participants in the scoped articles with only 62 out of the 525 participants being female. The performance gap disparity in endurance physiology is now being addressed in greater detail. For instance, observations in marathon running of better maintenance of pace in females, albeit it is unclear if there is a causal relationship between physiological sex-differences to explain this observation or whether psychological decision-making underlies this observation (Deaner et al. 2015). Moreover, sex differences exists in markers of endurance performance linked to neuromuscular fatigability (Billaut and Smith 2009; Ansdell et al. 2020). Combined with newly available observational data in women’s road-cycling races (Erp and Lamberts 2023) protocols to mimic the demands in this cohort should also be developed.

**Fig. 2:**
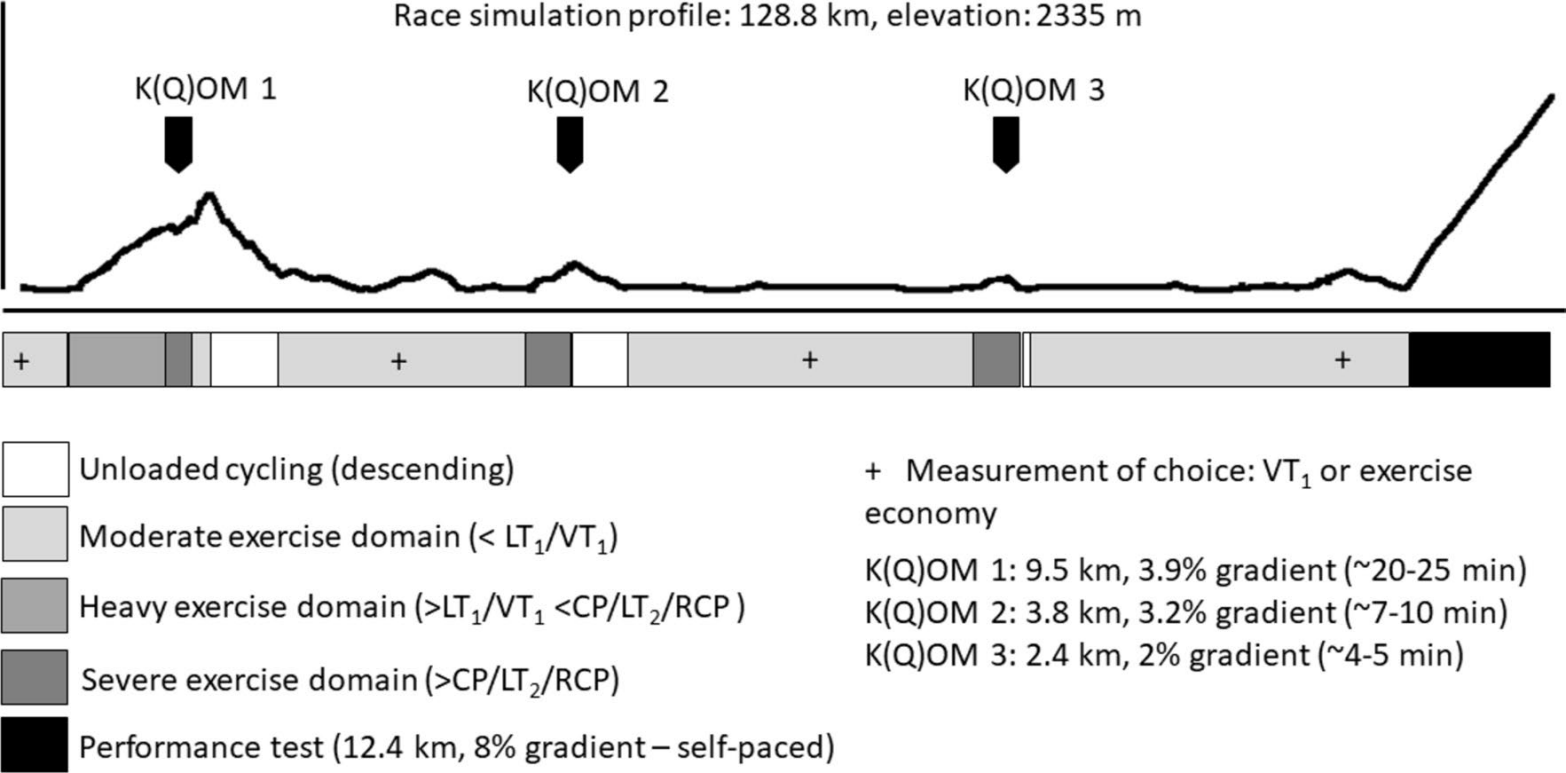
Example of a race simulation protocol utilising a subscription-based virtual cycling platform. The course is 128.8 km in length, or 116.4 km of pre-load followed by a 12.4-km uphill performance test. Proposed exercise intensities would mimic real-life road cycling race, with exercise intensity differing between longer-duration climbs (heavy), short-duration climbs (severe) and flat roads (moderate) or downhill (unloaded). The methodology benefits from real-time force feedback dictated by the terrain, incorporates the influence of body weight (W/kg) during uphill segments, and uses a fixed-distance approach which aligns with determining factors during outdoor races. At several points (+ as a suggestion) throughout the simulation, measurements (e.g., sub-maximal step-ramp test) could be incorporated to track markers influenced by fatigue resistance. K(Q)OM: King/Queen of the Mountain

One limitation with the current race simulation protocols is that alterations in power outputs during the pre-load are abrupt, step-wise in-, or decrements. Although some equipment and software’s can utilise and regulate real-time force feedback, thereby adjusting self-selected power output and as such simulate gradients (O'Brien et al. 2023; Clark et al. 2014), in recent years a digital and technological revolution has seen the creation of virtual cycling world applications. Connecting a “smart-trainer” to these virtual worlds enables cyclist to “ride up” climbs, which mimics the real-life experience and provide live resistance feedback depending on the gradient. To this extent, recent developments in online cycling applications might provide a novel tool to experiment with fixed-distance protocols on various virtual terrains that simulate outdoor cycling and account for the influence on body mass. An example of a protocol is presented in Fig. 2. Software applications such as Zwift™, Rouvy™ or MyWoosh™ allow users to meet up and race with fellow users which could enhance ecological validity if implemented in the research area. Reliability of the smart-trainer’s power output has been acceptable (Zadow et al. 2016) and some studies have already started using these virtual worlds in experiments (Montanari et al. 2023; Matta et al. 2022) whilst official E-sports competitions adopting these platforms too (Cheung 2021). Although further research is required, these developments have potential to create exciting opportunities as a method for scientific experiments into cycling race simulations.

## Conclusion

Research relating to determinants of road-cycling performance has entered renaissance with the introduction of the concept of physiological resilience. Whilst the mechanisms that influence one’s ability to withstand a decline in performance following accumulated work are yet to be fully elucidated, it is important that future experiments taking place inside the laboratory are implementing cycling protocols that mimic the demands of cycling road races, whilst taking into account the reliability of performance tests. To this extent, this review suggests that currently available cycling race simulation protocols present variability in meeting these principles, such as ecological validity, reliability and nutritional support, and researchers should select a protocol of the current list with caution or encourage them to develop protocols to the highest possible standard.

## Supplementary Information

Below is the link to the electronic supplementary material.

Supplementary file1 (DOCX 32 KB)

## Data Availability

The systematic search spreadsheet will be made available by the corresponding author upon reasonable request.

## Abbreviations

*BM*: Body mass
*CHO*: Carbohydrates
*CP*: Critical power
*CV*: Coefficient of variation
*HR*: Heart rate
*LT*: Lactate threshold
*PPO*: Peak power out
$\dot{V}$*O*_*2max*_: Maximal oxygen uptake capacity
*VT*: Ventilatory threshold
