# Supplementary material for: What is a cycling race simulation anyway: a review on protocols to assess durability in cycling
Source: Eur J Appl Physiol. 2025 Feb 14;125(6):1527–48. doi: 10.1007/s00421-025-05725-1 (PMC12174182; doi:10.1007/s00421-025-05725-1)
Supplement: Supplementary file 1 — Supplementary file1 (DOCX 32 KB) [file 421_2025_5725_MOESM1_ESM.docx]

***Supplemental Material 1***

***Searches***

The scoping review followed the Preferred Reporting Items for Systematic Reviews and Meta-Analyses – Scoping Review (PRISMA-ScR) guidelines [1], however the Population, Intervention, Comparison, Outcome (PICO) framework was adapted to better address the research question. More specifically, no comparison was used as the research question focussed on collating existing protocols regardless of which experimental comparison they were used for. The population of interest was healthy, active humans, the intervention was the preload and the outcome was the cycling performance test, which are further defined in the study inclusion criteria below. An initial search string was developed that used keywords “cycling” and “simulated race” or “preload” and “performance”. Inspecting several known articles and further discussion amongst reviewers (WP, MB, TP), more keywords were included to broaden the search. For example, specificity was included in the performance outcome (e.g. “time trial”, “sprint”) or specific, common durations of cycling interventions were included (e.g. “90-min”, “2-h”). A full search string with Boolean operators is presented in **Table 1**. Three databases (MEDLINE, Scopus and Web of Science) were accessed on the 9^th^ of January 2024 and all keywords were searched for in the title and abstract.

***2.2 Study inclusion criteria***

Only articles from peer-reviewed journals were included. Articles were included if the full-text was retrievable and published in English. The sample population had to be between 18 and 65 years old, consisting of healthy, active individuals, defined by having at least a measured $\dot{V}$O_2max_ > 40 or 45 ml/kg/min for males and females respectively. Studies with a mixed sample (e.g. trained vs. untrained) were included if they met other inclusion criteria. The exercise protocol had to be identified as laboratory-based and exclusively using cycling exercise, whereas protocols using multiple types of exercise were excluded (e.g. cycling and running). The cycling protocol in articles had to include at least a 90-min exercise protocol on a cycling ergometer (“pre-load”) followed by a cycling performance test. Justification for this lower time boundary was to assume a reasonable probability to accumulate significant amount of work to induce metabolic perturbations that could affect fatigue, such as glycogen depletion [2]. The nature of the pre-load protocol had to include a variable or stochastic intensity protocol. Thus, articles were excluded if the exercise prior to the performance test was a fixed-intensity protocol (e.g. 2h at 50% $\dot{V}$O_2max_). The reason for this was that the ecological validity of fixed-intensity protocol is limited compared to power output profiles of road cycling races [3]. Moreover, performance has been shown to be differentially affected following a stochastic exercise pre-load compared to continuous fixed-intensity [4, 5].

***2.3 Data extraction strategy***

Search results from the three databases were exported and stored in Microsoft Excel. Following the removal of duplicates, two reviewers (WMP and MB) independently screened the titles and abstracts against eligibility criteria. In the first round of title and abstract screening, inclusion was not filtered for the cycling protocol inclusion criteria in order to access full-text for inspection. The method and reference lists of the full-text were scanned for further articles. Discrepancies between the two reviewers were initially resolved by discussion. In case of no resolution, a third reviewer (TP) provided the final consensus.

***2.4 Data synthesis and presentation***

Two reviewers (WP, TP) extracted data from articles that met the inclusion criteria. Data was extracted on participant characteristics, equipment and environmental conditions, a breakdown of the preload protocol, the cycling performance test completed following the preload, nutritional considerations before and during the cycling and data on measures of reliability where available or origin for justification (e.g. based on field observations). In the case where a protocol was used by multiple studies, the reviewers traced back to the first publication using the protocol but presented which studies had used the protocol subsequently. Two reviewers (TP, WP) evaluated the strength of dietary control in relation to CHO intake using an adapted scoring framework from Close and colleagues [6]. Scores (-1, 0, +1) were assigned to qualitative and quantitative statements on dietary provision. For qualitative evaluation, -1 was assigned when no statement was present whether dietary intake was controlled, 0 was assigned if there was a statement on dietary control, but based on self-reported adherence or where advice-only was given. +1 was assigned if dietary control was present with participants receiving food products from the research team. For quantitative scores, -1 was assigned if no information on quantities was provided, 0 where quantities are stated but not in line with guidelines and +1 if quantities were stated and in line with guidelines. Scores were assigned for three feeding phases: 24-48 hours before a trial, hours before a trial and carbohydrate intake during the trial. Overall scores were ‘Inadequate’ (-6, -3), ‘Limited’ (-2, +2), or ‘Adequate’ (+3, +6). Guidelines used for quantitative scores for every phase were derived from Thomas *et al.* [7]: 1) carbohydrate intake of 10-12 g CHO per kg body mass per day 36-48 h before exercise, 2) 1-4 g CHO per kg body mass 1-4 h pre-event and 3) carbohydrate intake during exercise of 30-60 g per hour (1-2.5 hour events) and 60-90 g per hour (>2.5 hour events).

| Table 1. Search string as used in one of the three databases (MEDLINE). | |
| --- | --- |
| Intervention | (simulated cycling race[Title/Abstract] OR race simulation[Title/Abstract] OR simulated road cycling[Title/Abstract] OR distance[Title/abstract] OR preload[Title/Abstract] OR 90-min[Title/Abstract] OR 120-min[Title/Abstract] OR 180-min[Title/Abstract] OR 150-min[Title/Abstract] OR 105-min[Title/Abstract] OR 240-min[Title/Abstract] OR 2 h[Title/Abstract] OR 3 h[Title/Abstract] OR 4 h[Title/Abstract] OR fatiguing protocol[Title/Abstract] OR prolonged exercise[Title/Abstract]) |
| AND |  |
| Outcome | (performance[Title/Abstract] OR test[Title/Abstract] OR time trial[Title/Abstract] OR time to fatigue[Title/Abstract] OR time to task failure[Title/Abstract] OR time to exhaustion[Title/Abstract] OR sprint[Title/Abstract]) |
| AND |  |
| Specificity | Cycling[Title/Abstract] |

**References**

1. Tricco AC, Lillie E, Zarin W, O'Brien KK, Colquhoun H, Levac D, et al. PRISMA Extension for Scoping Reviews (PRISMA-ScR): Checklist and Explanation. Ann Intern Med. 2018;169(7):467-73. doi: 10.7326/m18-0850.

2. Hawley JA, Schabort EJ, Noakes TD, Dennis SC. Carbohydrate-loading and exercise performance. An update. Sports Med. 1997;24(2):73-81. doi: 10.2165/00007256-199724020-00001.

3. Sanders D, Heijboer M. Physical demands and power profile of different stage types within a cycling grand tour. Eur J Sport Sci. 2019;19(6):736-44. doi: 10.1080/17461391.2018.1554706.

4. Palmer GS, Noakes TD, Hawley JA. Effects of steady-state versus stochastic exercise on subsequent cycling performance. Med Sci Sports Exerc. 1997;29(5):684-7. doi: 10.1097/00005768-199705000-00015.

5. Leo P, Giorgi A, Spragg J, Gonzalez BM, Mujika I. Impact of prior accumulated work and intensity on power output in elite/international level road cyclists—a pilot study. German Journal of Exercise and Sport Research. 2022;52(4):673-7. doi: 10.1007/s12662-022-00818-x.

6. Close GL, Kasper AM, Morton JP. From Paper to Podium: Quantifying the Translational Potential of Performance Nutrition Research. Sports Med. 2019;49(Suppl 1):25-37. doi: 10.1007/s40279-018-1005-2.

7. Thomas DT, Erdman KA, Burke LM. Position of the Academy of Nutrition and Dietetics, Dietitians of Canada, and the American College of Sports Medicine: Nutrition and Athletic Performance. J Acad Nutr Diet. 2016;116(3):501-28. doi: 10.1016/j.jand.2015.12.006.
